# A comparison of severity of illness between the SARS-CoV-2 Omicron variant and Delta variant

**DOI:** 10.1017/ash.2023.453

**Published:** 2023-10-27

**Authors:** Laura Peyton Ellis, Olivia Hess, Khoa Le Anh Huynh, Gonzalo Bearman, Le Kang, Christopher D. Doern

**Affiliations:** 1 Obstetrics and Gynecology Residency Program, University of Connecticut, Farmington, CT, USA; 2 School of Medicine, Virginia Commonwealth University, Richmond, VA, USA; 3 Department of Biostatistics, Virginia Commonwealth University, Richmond, VA, USA; 4 Richard P. Wenzel Professor of Medicine, Chair, Division of Infectious Diseases, Virginia Commonwealth University, Richmond, VA, USA; 5 Antimicrobial Stewardship and Healthcare Epidemiology, Cambridge University Press, Cambridge, UK; 6 Department of Biostatistics, Virginia Commonwealth University, Richmond, VA, USA; 7 Microbiology & Pathology and Pediatrics, Virginia Commonwealth University, Richmond, VA, USA

## Abstract

**Background:**

The COVID-19 pandemic has disproportionally affected traditionally marginalized groups. Both the Delta and Omicron variants raised concern amongst public health officials due to potentially higher infectivity rates and disease severity than prior variants. This study sought to compare disease severity between adults infected with the Omicron variant and adults infected with the Delta variant who presented to the Emergency Department at an academic, safety-net hospital in Virginia.

**Methods:**

This retrospective cohort study used electronic medical record data of patients who presented to the Emergency Department and received a positive SARS-CoV-2 test between September 1, 2021, and January 31, 2022. Positive tests were stratified by genotypic variant through whole genome sequencing. Participants with the Omicron variant were propensity scores matched with individuals with the Delta variant.

**Results:**

Among 500 Delta and 500 Omicron participants, 279 propensity score-matched pairs were identified. Participants were predominantly unvaccinated, with medical comorbidities, and self-identified as Black. Individuals infected with the Delta variant had more severe disease compared to those with the Omicron variant, regardless of vaccination status. Patients with kidney, liver, and respiratory disease, as well as cancer, are at higher risk for severe disease. Patients with 2 doses of COVID-19 immunization trended toward less severe disease.

**Conclusions:**

Overall, these data further support the literature regarding the disproportionate effects of the COVID-19 pandemic on vulnerable patient populations – such as those with limited access to care, people of color, and those with chronic medical conditions – and can be used to inform public health interventions.

## Introduction

The World Health Organization (WHO) continues to collect data on variants of the wild-type novel coronavirus SARS-CoV-2. From many variants of interest and variants under monitoring, the WHO identified 5 variants of concern (VOCs) plaguing the international community in early 2022: Alpha, Beta, Gamma, Delta, and Omicron.^1^ Prior to the discovery of Omicron, the Delta variant was causing concern amongst public health officials as it was evidenced to be more infectious and generate a higher viral load than its predecessors.^2^ With respect to other VOCs, the less vaccinated an individual (unvaccinated, one dose, or <14 d after the second dose) the higher risk they were to develop severe disease. This trend was still generally true for the Delta variant, however, to a lesser degree.^3^ The Omicron variant accumulated additional mutations,^4^ namely in the spike protein, that further alter assay accuracy,^5^ infectivity,^6^ and vaccine effectiveness.^7^ Omicron is also capable of reinfecting individuals who have already developed antibodies through infection or vaccination, a concept called immune escape.^8,9^ One study found Omicron to be twice as infectious and twice as likely to escape vaccine immunity as the Delta variant.^10^ While whole genomic sequencing has provided insights as to how the structure and function of Omicron may be different from Delta, there has been less reported on differences in clinical severity. Potential illness severity is of particular importance to the patient population of urban Virginia considering widespread reports of higher morbidity and mortality in Black and Hispanic populations^11,12^ as well as communities with chronic medical conditions.^13^

This study aims to compare disease severity between adults infected with the Omicron variant as compared to adults infected with the Delta variant who presented to the Emergency Department at an academic center in Virginia.

## Methods

### Study center

This retrospective cohort study was conducted at a large, urban, academic, and safety net hospital in Virginia. As such, this hospital system receives patients from different geographic and socioeconomic statuses somewhat equally.

This study was approved by the Virginia Commonwealth University (VCU) Institutional Review Board and a waiver for informed consent was granted. No grant funding was used.

### Study participants

All patients age 18 and older presenting to the Emergency Department who received a positive SAR-CoV-2 test between September 1, 2021, and January 31, 2022, were eligible to participate. Positive tests were then stratified by genotypic variant through whole genome sequencing. The first 500 patients with Delta variant samples and the first 500 patients with Omicron variant samples were included in the study to minimize selection bias. Transfers from outside hospitals as well as samples from subsidiary hospitals were not included.

### Measures

The primary outcome of this study is illness severity as defined by both medical interventions and composite measures. All outcomes were collected via chart extraction by trained personnel (authors LPE, OH) for the duration of the index hospital stay related to COVID-19. Disease severity outcomes include symptom severity, non-ICU hospital admission, ICU admission, length of hospital stay, treatment (≥4 d remdesivir, ≥4 d corticosteroids, ≥1 dose convalescent plasma, ≥1 dose monoclonal antibody),^14^ supplemental oxygen requirement (nasal cannula), high flow oxygen requirement: (continuous positive airway pressure, bilevel positive airway pressure, high-flow nasal cannula), mechanical ventilation, and death. Composite measures are based on the WHO clinical severity criteria.^15^ *Mild-moderate illness* is defined as non-ICU hospital admission and/or supplemental oxygen requirement. *Severe illness* is defined as any of the following: ICU admission, high flow oxygen requirement, mechanical ventilation, and death.

The secondary outcomes involved stratifying patients by vaccination status. As the United States does not have a national vaccine registry, vaccination status, if available, was obtained from clinician notes.

### Laboratory methods

Patients were diagnosed with COVID-19 at the VCU Health System Microbiology Laboratory in Richmond, VA. The laboratory accepted anterior nasal or nasopharyngeal flocked swab specimens and used only polymerase chain reaction-based methods. Due to the high demand for testing and supply chain shortages, 3 separate diagnostic systems were utilized throughout the study. Validation studies performed by the laboratory confirmed that these systems performed equally for the detection of SARS-CoV-2. The diagnostic systems used at the time of the study were the ThermoFisher TaqPath COVID-19 Combo Kit (Thermo Fisher Scientific, Waltham, MA), The BioFire Respiratory Pathogen Panel 2.1 (BioMerieux, Marcy-l’Toile, France), and the Cepheid Xpert Xpress CoV-2/Flu/RSV *plus* (Cepheid, Sunnyvale, CA).

### Statistical analysis

The baseline characteristics of those infected with the Delta and Omicron COVID-19 variants were compared using the χ^2^ or Mann-Whitney test. For each individual with a confirmed Delta variant infection, we identified a propensity score-matched control individual with an Omicron variant infection. Propensity score matching was done based on demographic and clinical variables and vaccination status. A propensity score was estimated based on age, sex, diabetes mellitus, race, hypertension, coronary artery disease, kidney disease, respiratory disease, cancer, number of comorbidities, vaccination status (at time of infection), and body mass index (BMI). We performed nearest neighbor matching with distance estimates propensity scores using logistic regression. Furthermore, proportions of persons with each outcome were calculated and compared between those with the Delta and Omicron variants and stratified by vaccination status; 95% Confidence Intervals (CIs) were calculated to express the spread. Multivariable logistic regression was used to calculate the adjusted odds ratios (aORs) and 95% CIs for factors associated with primary and secondary outcomes. Significance was defined at 2-sided *P* < 0.05. All analyses were done by the Virginia Commonwealth Department of Biostatistics (author KH) using R, version 4.2.1.

## Results

We identified 500 individuals infected with the Delta variant and 500 individuals infected with the Omicron variant between September 1, 2021 and January 31, 2022. Out of this sample, participants were propensity-score matched for a total of 279 pairs. Demographic factors, except for vaccination status, were similar between groups (Table 1). The median age for those infected with the Delta variant was 49 years [18.0, 89.0], while the median age for those infected with the Omicron variant was 51 years [18.0, 99.0]. Out of the 279 individuals with the Delta variant, 114 (40.9%) were male and 165 (59.1%) were female. For the Omicron variant, 117 (41.95%) were male and 162 (58.1%) were female. Both Delta and Omicron populations were predominantly Black [Black 194 (69.5%), White 72 (25.8%), Other 12 (4.3%), Unknown 1 (0.4%); Black 191 (68.5%), White 70 (25.1%), Other 13 (4.7%), Unknown 5 (1.8%), respectively]. Among individuals with the Delta variant, 104 (37.3%) had no comorbidities, 72 (25.8%) had 1 comorbidity, 47 (16.8%) had 2 comorbidities, 40 (14.3%) had 3 comorbidities, 11 (3.9%) had 4 comorbidities, and 5 (1.85%) had 5 comorbidities. With regards to the Omicron variant, 98 (35.1%) had no comorbidities, 72 (28.5%) had 1 comorbidity, 49 (17.6%) had 2 comorbidities, 42 (15.1%) had 3 comorbidities, 14 (5.9%) had 4 comorbidities, and 4 (1.4%) had 5 comorbidities.

**Table 1.** Characteristics of persons infected with SARS-CoV-2 Delta and Omicron variants

	Number of participants, (%), [95% CI]		*P* value
	Delta variant (*n* = 279)	Omicron variant (*n* = 279)	
**Age**			
Median [Min, Max]	49.0 [18.0, 89.0]	51.0 [18.0, 99.0]	0.3193
**Sex**			
Male	114 (40.9%)	117 (41.9%)	0.8635
Female	165 (59.1%)	162 (58.1%)	
**Race**			
Black	194 (69.5%)	191 (68.5%)	0.4304
White	72 (25.8%)	70 (25.1%)	
Other	12 (4.3%)	13 (4.7%)	
Unknown	1 (0.4%)	5 (1.8%)	
**Body Mass Index**			
Underweight	11 (3.9%)	16 (5.7%)	0.7619
Normal	73 (26.2%)	71 (25.4%)	
Overweight	65 (23.3%)	68 (24.4%)	
Obese	130 (46.6%)	124 (44.4%)	
**Comorbidities**			
Diabetes mellitus	71 (25.4%)	66 (23.7%)	0.694
Hypertension	113 (40.5%)	121 (43.4%)	0.5482
Coronary artery disease	34 (12.2%)	35 (12.5%)	1
Kidney disease	46 (16.5%)	54 (19.4%)	0.4397
Cancer	16 (5.7%)	16 (5.7%)	1
Respiratory disease	54 (19.4%)	60 (21.5%)	0.5996
Total number of comorbidities			0.9807
0	104 (37.3%)	98 (35.1%)	
1	72 (25.8%)	72 (28.5%)	
2	47 (16.8%)	49 (17.6%)	
3	40 (14.3%)	42 (15.1%)	
4	11 (3.9%)	14 (5.9%)	
5	5 (1.8%)	4 (1.4%)	
**Vaccination status at time of infection**			
No doses	165 (59.1%)	124 (44.4%)	0.0008
>14 days after first dose	6 (2.2%)	4 (1.4%)	
>14 days after second dose	96 (34.4%)	121 (43.4%)	
>14 days after third dose	12 (4.3%)	30 (10.8%)	

The groups differed significantly regarding vaccination status (*p =* 0.0008). Those infected with the Delta variant were more likely to be unvaccinated against COVID-19 (59.1% vs 44.4%) compared to those with the Omicron variant. However, there was a negligible difference in the number of Delta patients who received 1 dose (2.2%) compared to the Omicron patients that received 1 dose (1.4%). Furthermore, individuals infected with Omicron were more likely to have 2 doses (43.4%) than their Delta counterparts (34.4%). This relationship was reflected for 3 doses as well, where 10.8% of Omicron patients had 3 doses compared to 4.3% of Delta patients. It is important to recognize the degree of missing vaccination status data (11% Delta, 17% Omicron) given the United States’ lack of a vaccine registry.

Next, groups were analyzed by COVID-19 variant (Table 2). Individuals infected with the Delta variant were more likely to be symptomatic (89.6% vs 78.1%, *p =* 0.0004), be admitted to the hospital (47.0% vs 29.0%, *p <* 0.0001), be admitted to the ICU (12.9% vs 5.73%, *p* = 0.0057), have a longer length of stay (7.73 d vs 8.55 d, *p* = 0.0038), require supplemental oxygen (34.4% vs 22.2%, *p* = 0.0019), and require high flow oxygen (15.4% vs 8.60%, *p* = 0.0191) compared to those infected with the Omicron variant. Individuals infected with the Delta variant were also more likely to have mild-moderate disease (33.7% vs 20.1%, *p* = 0.0004). Though there was no difference in rates of severe-critical disease, patients with the Delta variant were more likely to require treatment with corticosteroids (83, 29.7%, *p =* 0.0087) and monoclonal antibodies (80, 28.6%, *p =* <0.0001).

**Table 2.** Propensity score matched disease outcomes of persons infected with SARS-CoV-2 Delta and Omicron variants

	Number of participants, (%)		*P* value
	Delta variant (*n* = 279)	Omicron variant (*n* = 279)	
**Individual disease severity measures**			
Symptomatic	250 (89.6%)	218 (78.1%)	0.0004
Non-ICU hospitalization	131 (47.0%)	81 (29.0%)	<0.0001
ICU admission	36 (12.9%)	16 (5.73%)	0.0057
Length of stay – Mean (SD)	7.73 (7.37)	8.55 (6.79)	0.0038
Supplemental oxygen^a^	96 (34.4%)	62 (22.2%)	0.0019
High flow oxygen^b^	43 (15.4%)	24 (8.60%)	0.0191
Mechanical ventilation	17 (6.09%)	9 (3.22%)	0.1597
**Overall disease severity** ^c^			
Mild-Moderate	94 (33.7%)	56 (20.1%)	0.0004
Severe-Critical	43 (15.4%)	29 (10.4%)	0.1007
**Treatment**			
≥4 days Remdesivir	63 (22.5%)	56 (20.0%)	0.4694
≥4 days Corticosteroids	83 (29.7%)	57 (20.4%)	0.0087
≥1 dose of monoclonal antibodies	80 (28.6%)	15 (5.37%)	<0.0001

a ^.^Supplemental oxygen is defined as nasal cannula.

b High flow oxygen is defined as high flow nasal cannula, bilevel positive airway pressure, and continuous positive airway pressure.

c Mild-moderate disease included hospitalization and/or use of supplemental oxygen; severe-critical disease included any of the following: intensive care unit admission, use of high-flow oxygen, need for mechanical ventilation, and death.

Results were further stratified by variant and vaccination status (Table 3). Regardless of vaccination status, individuals with Delta variant were more likely to be admitted to the hospital (≤1 dose: 39.4% vs 22.6%, *p =* 0.0037; ≥ 2 doses: 57.9% vs 34.2%, *p =* 0.0001), require supplemental oxygen (≤1 dose: 31.5% vs 18.5%, *p =* 0.018; ≥ 2 doses: 38.6% vs 25.2%, *p=*0.026), and to meet criteria for mild-moderate disease (≤1 dose: 26.7% vs 12.9%, *p =* 0.006; ≥ 2 doses: 43.9% vs 25.85%, *p =* 0.002). Amongst patients who received 2 or more doses, those with Delta were more likely to be admitted to the ICU (15.8% vs 5.8%, *p =* 0.012). Conversely, those who received 2 or more doses and were then infected with the Omicron variant had longer lengths of stay (6.00 [0, 33.5] vs 4.68 [1.00, 36.5], *p =* 0.029.

**Table 3.** Clinical severity of persons infected with SARS-CoV-2 Delta and Omicron variants stratified by vaccination status

	One or fewer doses		*P* value	Two or more doses		*P* value
	*N* (%)			*N* (%)		
Variant	Delta (*N* = 165)	Omicron (*N* = 124)		Delta (*N* = 114)	Omicron (*N* = 155)	
**Individual disease severity measures**						
Non-ICU hospitalization	65 (39.4%)	28 (22.6%)	0.0037	66 (57.9%)	53 (34.2%)	0.0001
ICU admission	18 (10.9%)	7 (5.6%)	0.172	18 (15.8%)	9 (5.8%)	0.012
Length of stay (days)	6.63 [1.07, 35.6]	6.00 [2.00, 28.0]	0.186	4.68 [1.00, 36.5]	6.00 [0, 33.5]	0.029
Supplemental oxygen	52 (31.5%)	23 (18.5%)	0.018	44 (38.6%)	39 (25.2%)	0.026
High-flow oxygen	24 (14.5%)	11 (8.9%)	0.200	19 (16.7%)	13 (8.4%)	0.059
Mechanical ventilation	9 (5.5%)	2 (1.6%)	0.168	8 (7.0%)	7 (4.5%)	0.538
**Overall disease severity**						
Mild-Moderate	44 (26.7%)	16 (12.9%)	0.006	50 (43.9%)	40 (25.8%)	0.002
Severe-Critical	24 (14.5%)	14 (11.3%)	0.525	19 (16.7%)	15 (9.7%)	0.128

Lastly, factors potentially contributing to disease severity were investigated (Table 4). Patients infected with the Omicron variant were less likely to meet the criteria for mild-moderate disease Omicron [0.36 (0.23, 0.56) *p* < 0.0001], however, no effect was seen in the severe disease outcome. Race was not predictive of disease severity in either the mild-moderate group [Black 1, White 1.62 95% CI (1.01, 2.61) *p* = 0.0565, Other 1.57 95% CI (0.56, 4.44), *p* = 0.345, Unknown 2.39 95% CI (0.26, 21.76) *p* = 0.4451] or severe group [Black 1, White 0.91 95% CI (0.47, 1.77) *p* = 0.7959, Other 2.08 95% CI (0.54, 8) *p* = 0.2677, Unknown 2.23 95% CI (0.22, 22.87) *p* = 0.5456]. Hypertension [2.98 95% CI (1.92, 4.66) *p* < 0.0001] and kidney disease [2.12 95% CI (1.2, 3.78) *p* = 0.0101] contributed to the likelihood of contracting mild-moderate disease. Multiple comorbidities, including kidney disease [4.26 95% CI (1.97, 9.21) *p* < 0.0001, cancer [6.9 95% CI (2.53, 18.73) *p* < 0.0001], respiratory disease [5.78 95% CI (3.16, 10.59) *p* < 0.0001], and liver disease [3.91 95% CI (1.65, 9.3) *p* = 0.0022], greatly increased the risk of severe COVID-related outcomes. Those who received 2 vaccination doses were 55% less likely to have severe disease [95% CI (0.23, 0.88), *p* = 0.0102] compared to unvaccinated individuals. Conversely, those who received 3 vaccination doses were 3 times as likely to have mild-moderate disease [aOR: 3.2, 95% CI (1.43, 7.17) *p* = 0.0068] than their unvaccinated counterparts. Low BMI contributed to lower risk of mild-moderate disease [0.12 95% CI (0.01, 1.45) *p* = 0.0035], however, sample size was small (*n* = 27).

**Table 4.** Multivariable logistic regression with outcome disease status

	Mild-Moderate diseaseaOR, [95% CI]	*P* value	Severe diseaseaOR, [95% CI]	*P* value
**Variant**				
Delta	1 [Reference]		1 [Reference]	
Omicron	0.36 (0.23, 0.56)	<0.0001	0.57 (0.32, 1.01)	0.0687
**Race**				
Black	1 [Reference]		1 [Reference]	
White	1.62 (1.01, 2.61)	0.0565	0.91 (0.47, 1.77)	0.7959
Other	1.57 (0.56 4.44)	0.345	2.08 (0.54, 8)	0.2677
Unknown	2.39 (0.26, 21.76)	0.4451	2.23 (0.22, 22.87)	0.5456
**Comorbidities**				
Diabetes mellitus	1.06 (0.64, 1.75)	0.815	1.51 (0.78, 2.94)	0.1991
Hypertension	2.98 (1.92, 4.66)	<0.0001	1.32 (0.72, 2.44)	0.3933
Coronary artery disease	1.1 (0.6, 2.01)	0.7623	0.54 (0.23, 1.26)	0.1159
Kidney disease	2.12 (1.2, 3.78)	0.0101	4.26 (1.97, 9.21)	<0.0001
Cancer	1.55 (0.66, 3.6)	0.3538	6.9 (2.53, 18.73)	<0.0001
Respiratory disease	1.37 (0.84, 2.25)	0.2331	5.78 (3.16, 10.59)	<0.0001
Liver disease	0.61 (0.27, 1.38)	0.2656	3.91 (1.65, 9.3)	0.0022
**Vaccination status**				
None	1 [Reference]		1 [Reference]	
One dose	1 (0.23, 4.44)	0.9963	0 (N/A, N/A)	N/A
Two doses	1.24 (0.76, 1.99)	0.3716	0.45 (0.23, 0.88)	0.0102
Three doses	3.2 (1.43, 7.17)	0.0068	0.54 (0.18, 1.62)	0.2801
**BMI**				
Underweight	0.74 (0.23, 2.39)	0.6715	0.12 (0.01, 1.45)	0.0035
Overweight	0.62 (0.34, 1.15)	0.1298	0.89 (0.41, 1.92)	0.7486
Obesity	1.02 (0.61, 1.7)	0.9553	0.74 (0.37, 1.48)	0.3939

## Discussion

This study was a retrospective chart review using a stratified sampling method of Delta and Omicron variant infected patients who presented to an emergency department at an academic, safety-net hospital between September 2021 and January 2022. Analyses revealed worse overall disease outcomes and higher treatment requirements for patients infected with the Delta variant compared to propensity score-matched patients infected with the Omicron variant. Disease burden continued to be higher for Delta patients after stratifying by vaccination status. A multivariable model identified medical comorbidities such as kidney, liver, and respiratory disease along with cancer as predictors of more severe disease. Further, 2 doses of COVID-19 vaccine were reported to be protective against severe disease.

This study oversampled Black patients consistent with overall hospital demographics. As race is a social construct that is not representative of biological differences, it is important to analyze the ways structural racism has impacted the Black community throughout the COVID-19 pandemic. In New York City, the first epicenter of the pandemic, a higher population density of Black residents was the strongest predictor of neighborhood COVID-19 test positivity, even after controlling for socioeconomic status, comorbidities, and age.^16^ Later analyses of possible contributing factors reported that Blacks in New York City comprise a large sector of frontline jobs, increasing exposure to the virus.^17^ Another study found a decrease in the death rate in Black communities with higher unemployment, possibly due to ability to shelter in place.^18^ Racial/ethnic minorities as a whole are more likely to have positive COVID-19 test results and require admission to the hospital and ICU in the United States.^12,13,18,19^ This study was underpowered to detect inequities between racial groups, hence it is not surprising that race was not predictive of disease severity in the multivariable model. The study outcomes, however, largely represent a Black, urban patient population with unique vulnerabilities to the effects of COVID-19.

The COVID-19 literature has demonstrated that vaccination status plays a role in illness severity. Given the lack of standardized vaccine registry in the United States, the current study was not powered to detect a difference in disease severity by vaccination status. This study did, however, detect a trend toward protection from severe disease, regardless of variant, in patients who received 2 doses of the COVID-19 vaccine. Further, patients with 3 vaccine doses trended toward more mild-moderate disease compared to their unvaccinated counterparts. The study population who had received 3 doses was small (*n* = 42) and may represent a higher-risk group who was eligible for booster doses before the general public. Additionally, Black Americans have had the slowest uptake of the primary vaccine series and the second slowest uptake of the booster dose.^20^ Black participants in a survey conducted in the United States reported being the least likely racial/ethnic group to vaccinate themselves or those in their care, due to mistrust and perception of risk and severity.^21^ A large study conducted across American hospitals reported that 3 doses were necessary to achieve the same protection against the Omicron variant as 2 doses provided against the Delta variant.^22^ The authors note that in the unvaccinated population, the Delta variant drove higher disease severity. Therefore, the increased disease severity in the Delta variant population may be driven by higher proportion of unvaccinated and undervaccinated individuals. Furthermore, the effect of Delta on disease severity existed after controlling for vaccination status.

Disease severity was further compounded by medical comorbidities, which also display unsettling patterns of racial inequity. Patients with preexisting kidney disease and hypertension were roughly 2 and 3 times as likely to develop mild-moderate disease, respectively. Effects were greater in the severe disease cohort. Patients with kidney disease (aOR: 4.26), cancer (aOR: 6.9), respiratory disease (aOR: 5.78), and/or liver disease (aOR: 3.91) were at a substantially higher risk of suffering from severe disease outcomes, like ICU admission or requiring high-flow oxygen. The health inequity literature acknowledges several important trends in the COVID-19 pandemic. In a Black, urban population, participants were more likely to have hypertension, diabetes, and a higher number of comorbidities overall, regardless of age.^17^ Black patients are also over-represented in the asthmatic population, leading to more hospitalization secondary to COVID-19.^23^ Despite reported differences in baseline comorbidity rates, Black participants are underrepresented in COVID-19 prevention strategy trials.^24^ There is, however, equal representation of Black Americans in COVID-19 treatment trials.

This study has several strengths that add to the existing literature. Namely, a lower socioeconomic status, Black, and urban population were oversampled. Though insurance information was not readily available for all participants, it is reasonable to extrapolate socioeconomic status given typical hospital demographics, especially in the Emergency Department. Therefore, this study captures a particularly vulnerable population. The study time period was carefully selected to include the peak of both Delta and Omicron waves at the institution. The trends presented here regarding comorbidities, vaccination status, and disease outcomes inform the national discourse on how to protect a disproportionately affected sector of the United States population in the setting of another potential surge.

This study is limited in its generalizability. It was conducted at a single academic center at a cross-section in time. Vaccination status was based on patient report and provider documentation, which limits reliability and contributes to missing data. To control variables, this study evaluated only patients who presented to the emergency department and may be more symptomatic with greater numbers of comorbidities than other patient populations. However, it is the anecdotal observation of the authors during chart review that the ED was being used inappropriately by patients as an asymptomatic testing center at this stage of the pandemic. It has also been reported that Black patients are more likely to get COVID-19 testing in the ED or inpatient than in the ambulatory setting.^25^

Overall, these data support reports of higher morbidity due to the COVID-19 pandemic in minority populations and those suffering from chronic medical conditions. Disease severity was worse in the Delta variant cohort regardless of vaccination status. Illness severity was further mediated by medical comorbidities. This work serves to inform public health efforts to serve especially vulnerable patient populations with multiple medical comorbidities, such as the low socioeconomic status, predominantly Black urban population described here.
